# Error in Byline

**DOI:** 10.1001/jamanetworkopen.2026.34664

**Published:** 2026-08-21

In the Original Investigation titled “Prenatal Azithromycin Exposure and Risk of Neurodevelopmental Disorders in Children”^1^ published May 12, 2026, there was an error in Dong Keon Yon’s name in the byline. This article has been corrected.^1^
